# Effects of physical and social environments on the risk of dementia among Taiwanese older adults: a population-based case-control study

**DOI:** 10.1186/s12877-020-01624-6

**Published:** 2020-06-26

**Authors:** Chih-Ching Liu, Yu Sun, Shiann-Far Kung, Hsien-Wen Kuo, Nuan-Ching Huang, Chung-Yi Li, Susan C. Hu

**Affiliations:** 1 Department of Healthcare Administration, College of Medical and Health Science, Asia University, No. 500, Lioufeng Road, Wufeng District, Taichung, 41354 Taiwan; 2 Department of Neurology, En Chu Kong Hospital, No. 399, Fuxing Road, Sanxia District, New Taipei City, 23702 Taiwan; 3 Department of Urban Planning, College of Planning & Design, National Cheng Kung University, No.1, University Road, Tainan City, 701 Taiwan; 4 Healthy Cities Research Center, Research and Services Headquarters, National Cheng Kung University, No.1, University Road, Tainan City, 701 Taiwan; 5 Institute of Environmental and Occupational Health Sciences, School of Medicine, National Yang-Ming University, No.155, Sec.2, Linong Street, Taipei city, 112 Taiwan; 6 Department of Public Health, College of Medicine, National Cheng Kung University, No.1, University Road, Tainan City, 701 Taiwan; 7 Department of Public Health, College of Public Health, China Medical University, Taichung, 404 Taiwan

**Keywords:** Physical environments, Social environments, Dementia, Case-control studies, Incidence, Older adults

## Abstract

**Background:**

Physical and social environments may influence cognition health in older adults. However, evidence regarding physical and social environments linked to dementia is lacking, especially in Asia. This study aims to explore the influence of physical and social environments on the incidence of dementia through a population-based case-control design in Taiwan.

**Methods:**

We identified 26,206 incident cases with dementia aged≧65 years in 2010, with the same no. of controls from National Health Insurance claims. Environmental measures were collected from government statistics including three physical environments and three social environments. Multilevel logistic regression was used to estimate the odds ratios (ORs) and 95% confidence intervals (CI) of the association between dementia incidence and the environmental measures at the township level.

**Results:**

We observed a significant reduction of 12% in the odds ratios of dementia in areas with higher availability of playgrounds and sport venues (OR 0.88, 95% CI 0.81–0.95), after controlling for individual and other environmental characteristics. Community center availability was also significantly associated with an 8% decreased odds for dementia (OR 0.92, 95% CI 0.87–0.99), but the association was not significant after further consideration of individual-level characteristics. Although higher odds of dementia were found in areas with high median annual family income (OR 1.14, 95% CI 1.04–1.25), such a significant relationship did not appear in the full model.

**Conclusions:**

Our study suggests that specific physical and social environmental features have different influences on the risk of dementia. Public health interventions may consider these environmental aspects for preventing dementia incidence.

## Background

Dementia has been regarded as a public health challenge [1], as it is related to increased cost, potential disability and mortality [1, 2]. Identifying the risk or protective factors of dementia may thus represent an important issue in public health. Although individual factors such as education, lifestyle (social interaction, physical activity), and chronic diseases (vascular disease, diabetes mellitus, and depression) are important to the progression of dementia [3], other environmental factors may also play an important role in the risk of dementia [4, 5]. Some previous studies have suggested that certain features of both physical and social environments on a small scale, including the presence of community resources [6, 7], public open spaces [6, 7], green environments [7–9], neighborhood social cohesion [10], and socioeconomic composition of residential population [11–13], were associated with individual-level cognition function among elderly adults. Those features of physical and social environments may change the risk of dementia by moderating individual risk factors such as lifestyle [14, 15], chronic diseases [16–18], and by playing a potential role to enable or obstruct cognition stimulation [4] and cognition reserve [19].

Nevertheless, very few studies have investigated the relationships between the characteristics of physical or social environments in small areas and the risk of developing dementia, especially in Asia [7, 8, 20–23]. To the best of our knowledge, the only two study to date on this topic in Asia have been conducted in Japan [22] and Hong Kong [23]. One study from Japan used a cohort design with a 3-year follow-up period and found Geographic Information System (GIS)-based and self-reported living in neighborhoods with lower availability of food stores were associated with increased dementia incidence in older adults after controlling for individual features and other environmental factors, with an adjusted hazard ratio (HR) of 1.51 (95% CI 1.34–1.69) and 1.65 (95% CI 1.40–1.93), respectively [22]. However, this study focused more on food environments but with limited social environments, which may preclude meaningful information. Another study from Hong Kong explored the effect of small area-based physical and social environments on the risk of dementia simultaneously and reported neighborhoods with higher economic disadvantage (OR = 1.02, 95%CI 1.01–1.02), higher walkability (OR = 0.99, 95%CI 0.98–0.99), higher library accessibility (OR = 0.99, 95%CI 0.99–0.99) were associated with the risk of dementia after controlling for individual characteristics and other environmental factors [23]. However, this study, like most of other studies, has been limited by the relatively small number of dementia cases [7, 8, 20, 21] and its cross-sectional design [7, 8]. This not only makes it difficult to interpret the study findings but also precludes any firm conclusions.

To overcome the aforementioned methodological problems and limited information on environmental factor, we conducted this large-scale population-based case-control study in Taiwan to simultaneously assess the effects of three physical environments (parks, greeneries, and square area; playgrounds and sport venues; community centers) and three social environments (median annual family income, percentage of illiterate people aged≧65, density of elderly living alone) at the township level on the risk of developing dementia among elderly adults in Taiwan.

## Methods

### Study design and data source

This was a population-based case-control study linked to three national datasets: Taiwan’s National Health Insurance Research Data (NHIRD), the Age-Friendly Environment Database, and the 2006 National Land Use Investigation.

Individual-level data in this study were collected from three parts of the NHIRD, including ambulatory care claims, inpatient claims, and the updated registry for beneficiaries, which were provided by the National Health Insurance Administration (NHIA), Ministry of Health and Welfare, Taiwan. Access to the research data in the NHIRD was approved by the National Health Research Institutes Review Committee. Approved code by the National Health Research Institutes Review Committee: NHIRD-101-565. All methods were performed in accordance with the institution’s relevant guidelines and regulations. Informed consent for study participant was waived because personal identification numbers in the NHIRD are encrypted.

Information of ecological physical and social environments was retrieved from the Taiwan Age-Friendly Environment Database at the township level provided by Hu et al. (2018) [24]. Ecological data on the types of land use also at the township level, including “parks, greeneries, and square area” and “playgrounds and sport venues”, were derived from the earliest released national version in 2006 National Land Use Investigation, using the National Geographic Information System (GIS) in Taiwan.

### Selection of cases and controls

In this case-control study, we included patients aged 65 years and older who had at least three outpatient claim records of dementia-related diagnosis codes (International Classification of Disease, 9th Revision, Clinical Modification [ICD-9-CM] code of 290, 291, 294, 331, 046.1) after a first-time diagnosis of dementia in 2010, based on the NHIRD. This definition was based on the previous study on dementia using Taiwan’s NHIRD [25]. The first and last outpatient visits among these patients in 2010–2011 were separated by at least 90 days to avoid accidental inclusion of miscoded patients. We linked these dementia patients to the outpatient claim records from 2003 to 2009 to exclude those who obtained a dementia diagnosis prior to 2010 to confirm that only subjects with an initial diagnosis of dementia were included in our study (i.e., incident cases). The date of initial diagnosis for dementia was based on the first day of the dementia diagnosis in 2010 (referred to as the index date). Additionally, those patients who had moved to long-term care institutions before the index date were also excluded because they might have had different interactions with their living environments. Moreover, dementia cases with missing information on living area in 2006 were also excluded. In the end, 26,206 eligible dementia cases were identified.

**Fig. 1:**
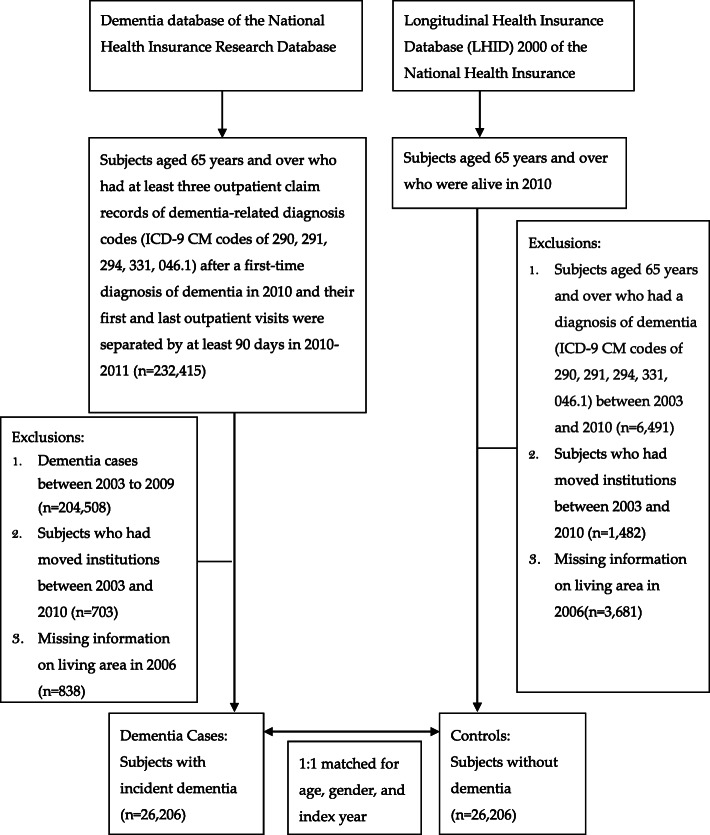
Flow chart describing the enrolment of study participants in the dementia and control groups

As for the control subjects, we recruited subjects aged 65 years and older who were alive in 2010. We excluded older adults who had a diagnosis of dementia (ICD-9 CM codes of 290, 291, 294, 331, 046.1) between 2003 and 2010. In addition, those who had moved to long-term care institutions from 2003 to 2010 or those who had missing information on living area in 2006 were also excluded. We then randomly selected the same number of control subjects as dementia cases by frequency matching on age, sex, and index year of dementia diagnosis (Fig. 1).

We determined the environmental exposure of our study subjects based on the township where he/she lived. Each study subject’s township was obtained from the Taiwan’s NHIRD records. The average size of townships was 98.33 km^2^ and the average population of townships were 62,164 (0.1–41.25 thousand people /per km^2^) in 2006 [26].

### Assessment of physical and social environments

First, we selected some physical and social environmental variables for the analysis, which were also analyzed in previous research [5, 7, 8, 20–22, 27]. Given that past studies on the association between physical and social environments and the risk of dementia were limited, and physical and social environmental variables were either diversely defined [7, 8, 20–22], we determined the environmental indicators for our analysis through several expert meetings until a consensus was reached by our research team and experts. Finally, three physical environments at the township level, potentially related to the risk of dementia, were assessed in this study, including (1) parks, greeneries, and square area, (2) playgrounds and sport venues, and (3) community centers. We computed the density of parks, greeneries, and square area (in km^2^ per 10^5^ people) to indicate the availability of green environments. Likewise, we calculated the density of playgrounds and sport venues (in km^2^ per 10^5^ people) to represent the availability of recreational resources. The areas were derived from the 2006 National Land Use Investigation and were intersected and calculated by ArcGIS (ArcMap, version 10.3; ESRI Inc., Redlands, CA, USA). The area in each township was calculated as per capita area (km^2^). Moreover, the density of community centers (no. of community centers/ per 10^5^ people) was derived from government statistics and was used to reflect the resources of community activity spaces.

Similarly, three indicators of social environments were also examined in this study from the Age-Friendly Environment Database, including (1) median annual family income, (2) percentage of illiterate people aged≧65, and (3) density of elderly living alone (number per 10^3^ elderly people). Based on the previous studies indicating that living alone was associated with an elevated prevalence of social isolation [28] and decreased social support [29], we hypothesized that areas with a higher density of elderly living alone may have less social cohesion. Furthermore, we used “median annual family income” and “percentage of illiterate people aged≧65” to indicate socioeconomic status and educational status, respectively.

### Assessment of potential confounders

For ecological covariates, the density of hospitals and clinics (no. of hospitals and clinics per 10^3^ elderly people) in each township was added into the model as a covariate to reduce the likelihood that the chance of being diagnosed as dementia could be due to different accessibility of medical resources. This indicator was obtained from the open data of the Taiwan Medical Association in 2006 (http://www.tma.tw/stats/index_AllPDF.asp). Levels of urbanization (urban, suburban, and rural) were also considered in the analysis to account for the urban-rural differences in accessibility and availability of medical care [30, 31]. The level of urbanization used in this study included 4 indicators: no. of residents, percentage of people working in secondary industries, percentage of people working in tertiary industries, and population density [32].

Individual-level potential confounders included age, sex (male and female), occupation, salary-based insurance premium, and number of comorbidities. Particularly, occupational status (white collar, blue collar, and others) and salary-based insurance premium (dependent, <median insurance premium, and ≧median insurance premium) were considered to adjust for the possible educational and socioeconomic difference among individuals, respectively [3]. Certain clinical risk factors, which may be associated with an increased risk of dementia, included hypertension, diabetes, heart disease (i.e., coronary artery disease or congestive heart failure), stroke, head injury, hyperlipidemia, depression, and chronic obstructive pulmonary disease (COPD) were also considered [3]. Information of selected comorbidities was retrieved from medical claims between 2003 and 2005. Only comorbidity that appears at least 3 times in outpatient claims or > 1 in inpatient claims within 1 year was counted.

**Fig. 2:**
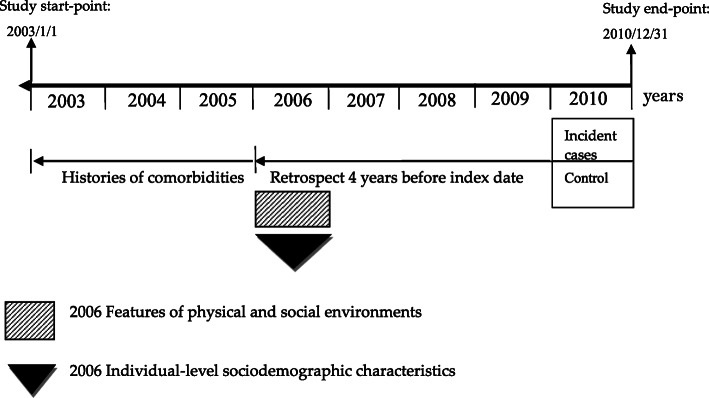
Timeline of exposure to physical and social environments or covariates in the dementia and control groups

In this study, we investigated each subject’s exposure to the physical and social environments in 2006 before the index date in order to capture the environmental characteristics within a township which may be associated with dementia incidence after adjustment of covariates. Figure 2 shows the diagram describing exposure time to physical and social environments or covariates in dementia cases and the control group.

### Statistical analysis

We first compared the differences in characteristics between dementia cases and controls. Descriptive statistics of physical and social environments at the residential township for the year 2006 were also conducted. Since the distributions of environmental characteristics in this study were skewed, we categorized the environmental measures into tertiles (the lowest served as the reference group) and examined their relationships with the risk of dementia.

Two-level random intercept logistic regression models were performed to examine the odds ratios (ORs) and 95% confidence intervals (CIs) for the relationship between the features of both physical and social environments and the risk of dementia. The SAS procedure GLIMMIX was adopted for fitting multilevel logistic regression models (individual at level 1 nested within townships/cities at level 2) with a binary outcome and a *logit* link [33].

To account for variations in dementia risk between townships, we first used the null model which did not include any predictors to examine the error variance of the level-2 intercept (Model 0). Second, features of physical environments were entered into the null model to predict dementia risk (Model 1). Third, features of social environments were added to the null model to examine the association between the social environments and dementia risk (Model 2). Fourth, features of both physical and social environments were simultaneously included into the model to investigate combination effect of both physical and social environments on the risk of dementia (Model 3), in which individual characteristics were adjusted in Model 4, then individual characteristics, hospitals and clinics, and urbanization status were adjusted in Model 5. The variance inflation factor (VIF) of each variable was computed to detect the potential multicollinearity among physical and social environments and covariates in the regression model.

All VIF in the above models were less than 3, which shows little multicollinearity of the models. The statistical analysis was performed by SAS version 9.4 (SAS Institute, Cary, NC, USA). A *P*-value of < 0.05 was considered statistically significant.

## Results

### Study population of dementia and controls

Table 1 shows that the distributions of gender and age in both dementia cases and the control group were comparable. However, there were fewer white-collar workers (11.8% vs. 12.7%) among the dementia patients and they were more dependent (37.3% vs. 36.9%), and had more comorbidities (30.3% vs. 22.9%) than the control group.

**Table 1:** Individual characteristics of dementia cases and controls

Variables^a^	Dementia		Control		*p*
	n	%	n	%	
**Individual characteristics**					
Gender					1.0000^e^
Men	11,873	45.3	11,873	45.3	
Female	14,333	54.7	14,333	54.7	
Age (years)					1.0000^e^
65–69	2496	9.5	2496	9.5	
70–74	4614	17.6	4614	17.6	
75–79	6078	23.2	6078	23.2	
80–84	6813	26.0	6813	26.0	
≧80	6205	23.7	6205	23.7	
Mean ± SD^b^	79.3 ± 7.0		79.0 ± 7.0		
Occupational status					<.0001^e^
white collar	3095	11.8	3320	12.7	
blue collar	9839	37.5	10,332	39.4	
others	13,272	50.7	12,554	47.9	
Salary-based insurance premium (NTD)^b^					<.0001^e^
dependent	9714	37.3	9667	36.9	
< Median (19,200)	6673	25.6	6153	23.5	
≧Median	9679	37.1	10,386	39.6	
Mean ± SD^b,c^	8095.0 ± 11,038.5		8736.0 ± 11,752.3		
Number of comorbidities ^d^					<.0001^e^
0	6058	23.1	7885	30.1	
1–2	12,290	46.9	12,334	47.1	
≧ 3	7858	30.0	5987	22.9	
Total	26,206	100.0	26,206	100.0	

^a^ Inconsistency between total population and population summed for individual variables was due to missing information

^b^*SD* Standard deviation, *NTD* New Taiwan Dollars

^c^ The dependent insurers were not included

^d^ Number of comorbidities included hypertension, diabetes, heart disease (i.e., coronary artery disease or congestive heart failure), stroke, head injury, hyperlipidemia, depression, and chronic obstructive pulmonary disease

^e^ Based on χ^2^ test for category variables

### Descriptive statistics of dementia in relation to environmental exposure density in the residential township

Table 2 shows that the distributions of the characteristics of physical and social environments in the 349 residential townships of the study subjects for the year 2006 are highly skewed. Thus, these environmental characteristics were stratified into tertiles based on their distribution in 2006 and were presented according to dementia cases and controls (Table 3). The dementia cases (*n* = 26,206) and controls (n = 26,206) were nested within the 343 and 344 townships/cities, respectively.

**Table 2:** Descriptive statistics of environmental exposure in the residential township (*n* = 349) of the study subjects

Environmental features	Percentile					
	Min	Max	Mean ± SD^a^	Median	33th	66th
**Physical environments**						
Parks, greeneries, and square area^b^	0	25.98	0.54 ± 1.50	0.29	0.17	0.42
Playgrounds and sport venues^b^	0	156.23	1.42 ± 8.58	0.21	0.11	0.46
Community centers^c^	0	324.75	42.68 ± 43.45	31.90	15.28	49.13
**Social environments**						
Median annual family income^d^	339	829	507.24 ± 68.02	494	473	519
Illiterate people aged≧65 (%)	0.22	55.93	18.69 ± 11.04	16.29	12.65	22.21
Elderly living alone^e^	0	539.29	38.91 ± 51.74	19.15	13.72	32.26

^a^*SD* Standard deviation

^b^ km^2^/per10^5^ people

^c^ number/per 10^5^ people

^d^ 1000 New Taiwan Dollars

^e^ number / per10^3^ elderly people

**Table 3:** Features of living environments in dementia cases and controls

Variables^a^	No. of townships (Dementia/control)	Dementia		Control		*p*^g^
		n	%	n	%	
**Physical environments**						
Parks, greeneries, and square area^a,b^						0.1368
Low	112/112	8101	30.9	8157	31.1	
Medium	119/118	10,982	41.9	11,126	42.5	
High	112/114	7123	27.2	6923	26.4	
Playgrounds and sport venues^a,b^						<.0001
Low	116/115	13,177	50.3	12,815	48.9	
Medium	113/114	9043	34.5	9046	34.5	
High	114/115	3986	15.2	4345	16.6	
Community centers ^a,c^						<.0001
Low	116/115	18,104	69.1	17,580	67.1	
Medium	115/113	5818	22.2	6205	23.7	
High	112/116	2284	8.7	2421	9.2	
**Social environments**						
Median annual family income^a,d^						0.0002
Low	106/109	2521	9.6	2796	10.7	
Medium	116/115	6029	23.0	6067	23.2	
High	121/120	17,656	67.4	17,343	66.1	
Illiterate people aged≧65^a,e^						0.0364
Low	109/110	11,697	44.6	11,558	44.1	
Medium	116/116	9746	37.2	9656	36.9	
High	118/118	4763	18.2	4992	19.0	
Elderly living alone^a,f^						0.7884
Low	116/116	12,348	47.1	12,345	47.1	
Medium	113/113	9575	36.5	9630	36.8	
High	114/115	4283	16.4	4231	16.1	
**Other environments**						
Hospitals and clinics^a,f^						0.0005
Low	113/115	2338	8.9	2549	9.7	
Medium	111/109	5581	21.3	5741	21.9	
High	119/120	18,287	69.8	17,916	68.4	
Urbanization						<.0001
Rural	201/202	6352	24.2	6929	26.5	
Suburban	114/114	12,612	48.1	12,193	46.5	
Urban	28/28	7242	27.6	7084	27.0	
Total	343/344	26,206	100.0	26,206	100.0	

^a^ Low: < 33%, Median: 34–65%; High: ≥ 66%

^b^ km^2^/per 10^5^ people

^c^ number/per 10^5^ people

^d^ 1000 New Taiwan Dollars

^e^ %

^f^ number/ per10^3^ elderly people

^g^ Based on χ^2^ test

The characteristics of “parks, greeneries, and square area” and “elderly living alone” were similar for the dementia and control groups. The percentages of dementia patients who lived in the highest tertile of playgrounds and sport venues (15.2% vs. 16.6%), and in the highest tertile of community centers (8.7% vs. 9.2%) were lower than those of their counterparts. In addition, patients with dementia were more likely to live in townships with higher median annual family income (percentage with the highest tertile of median annual family income: 67.4% vs. 66.1%), less illiterate people aged≧65 (percentage with the highest tertile of illiterate people aged≧65: 18.2% vs. 19.0%), more hospitals and clinics (percentage with the highest tertile of hospitals and clinics: 69.8% vs. 68.4%), and more likely to live in urban (27.6% vs. 27.0%) and suburban (48.1% vs. 46.5%) areas (Table 3).

### Odds ratio of dementia in relation to individual characteristics and environmental exposure density

Table 4 reveals the results of multilevel logistic regression modeling. The null model showed the risk of dementia varied significantly across townships (error variance level-2 intercept: 0.02685, *p* < .0001), indicating that it is necessary to take into account township-level variables in the analysis. Model 1 showed that cases were less likely than controls to live in townships with a high density of playgrounds and sport venues (OR = 0.88, 95% CI 0.82–0.95). Similarly, compared with controls, cases had a significantly lower prevalence of living in townships with a medium density of community centers (OR = 0.92, 95%CI 0.87–0.99). Model 2 showed that cases were more likely than controls to live in townships with medium and high median annual family incomes (OR = 1.13, 95% CI 1.04–1.23 and OR = 1.14, 95% CI 1.04–1.25, respectively). Considering physical and social environments simultaneously, compared with controls, cases still had a significantly lower prevalence of living in townships with a high density of playgrounds and sport venues and a medium density of community centers (Model 3). However, the significant association between high median annual family income and dementia risk did not appear. After further controlling for individual characteristics, only a high density of playgrounds and sport venues (OR = 0.88, 95% CI 0.82–0.96) and medium median annual family income (OR = 1.11, 95% CI 1.01–1.21) were still significantly associated with the risk of dementia (Model 4). Such an inverse relationship between the density of playgrounds and sport venues and the risk of dementia remained unchanged after adjustment for individual characteristics, hospitals and clinics, and urbanization status (OR = 0.88, 95% CI 0.81–0.95) (Model 5). Again, we observed null association between living in townships with a medium median annual family income and dementia in Model 5 with adjustment of individual characteristics, hospitals and clinics, and urbanization status (OR = 1.09, 95% CI 0.99–1.19).

**Table 4:** Odds ratio of dementia in relation to individual characteristics and environmental exposure in the residential township of the study subjects (Environmental exposure according to tertile classification)

Variables	Model 1	Model 2	Model 3	Model 4	Model 5
**Intercept (SD)**^a^	0.05 (0.03)	−0.16* (0.05)	−0.06 (0.06)	−0.30* (0.06)	−0.34* (0.08)
**Physical environments**					
Parks, greeneries, and square area (ref: Low)^b^					
Medium	0.98 (0.92–1.04)		0.98 (0.91–1.04)	0.97 (0.91–1.04)	0.97 (0.91–1.04)
High	1.02 (0.95–1.09)		1.02 (0.95–1.09)	1.01 (0.94–1.09)	1.00 (0.93–1.08)
Playgrounds and sport venues (ref: Low)^b^					
Medium	0.98 (0.92–1.05)		0.98 (0.92–1.04)	0.98 (0.92–1.05)	0.97 (0.90–1.04)
High	0.88* (0.82–0.95)		0.88* (0.81–0.95)	0.88* (0.82–0.96)	0.88* (0.81–0.95)
Community center (ref: Low)^b^					
Medium	0.92*(0.87–0.99)		0.92*(0.86–0.99)	0.94 (0.87–1.00)	0.95 (0.88–1.02)
High	0.94 (0.86–1.02)		0.94 (0.86–1.04)	0.95 (0.86–1.05)	0.98 (0.88–1.08)
**Social environments**					
Median annual family income (ref: Low)^b^					
Medium		1.13* (1.04–1.23)	1.12* (1.03–1.22)	1.11* (1.01–1.21)	1.09 (0.99–1.19)
High		1.14*(1.04–1.25)	1.08 (0.98–1.19)	1.04 (0.94–1.15)	1.01 (0.91–1.12)
Illiterate people aged≧65 (ref: Low)^b^					
Medium		1.04 (0.97–1.13)	1.04 (0.97–1.12)	1.05 (0.98–1.12)	1.04 (0.97–1.12)
High		1.02 (0.94–1.11)	1.03 (0.95–1.12)	1.05 (0.96–1.15)	1.06 (0.97–1.15)
Elderly living alone (ref: Low)^b^					
Medium		1.00 (0.93–1.06)	1.01 (0.95–1.07)	1.01 (0.94–1.07)	1.01 (0.95–1.08)
High		1.05 (0.97–1.14)	1.06 (0.98–1.15)	1.07 (0.99–1.16)	1.08 (0.99–1.18)

In the null model, the estimated error variance level-2 Intercept (SD) was also 0.02685 (0.005013), *p* < .0001

^a^*SD* Standard deviation

^b^ Low: < 33%, Medium: 34–65%; High: ≥ 66%

Model 3: Model 1 + Model 2

Model 4: Model 1 + Model 2+ individual variables (insurance premium, occupational status, and number of comorbidities)

Model 5: Model 4+ hospitals and clinics, and urbanization status

* *p* < 0.05

In Table 5, we further compared higher availability of playgrounds and sport venues with annual family income to test for a significant increase or reduction in the risk of developing dementia. We noted that cases were more likely than controls to live in townships with medium and high median annual family incomes (OR = 1.24, 95% CI 1.05–1.46 and OR = 1.26, 95% CI 1.04–1.52, respectively) in Model 1 with adjustment of illiterate people aged≧65 and elderly living alone. However, when we considered physical and social environments simultaneously, the significant association between high median annual family income and dementia risk did not appear (Model 2). After further controlling for individual characteristics, medium median annual family income (OR = 1.18, 95% CI 1.00–1.40) was still significantly associated with the risk of dementia (Model 3). Again, we observed a null association between living in townships with a medium median annual family income and dementia in Model 4 with adjustment of individual characteristics, hospitals and clinics, and urbanization status (OR = 1.16, 95% CI 0.98–1.39). These results (Model 1–4) were similar to the findings based on the whole study sample.

**Table 5:** Odds ratio of dementia in relation to higher availability of playgrounds and sport venues according to median annual family income

Variables	Model 1	Model 2	Model 3	Model 4
Higher availability of playgrounds and sport venues/Lower median annual family income^a^	1.00	1.00	1.00	1.00
Higher availability of playgrounds and sport venues/ Medium median annual family income^a^	1.24* (1.05–1.46)	1.20* (1.01–1.42)	1.18 * (1.00–1.40)	1.16 (0.98–1.39)
Higher availability of playgrounds and sport venues/ Higher median annual family income^a^	1.26* (1.04–1.52)	1.16 (0.95–1.42)	1.11 (0.90–1.36)	1.08 (0.88–1.32)

Model 1: Based on multilevel logistic regression and adjusted for illiterate people aged≧65, elderly living alone

Model 2: Model 1+ parks and greeneries, community centers

Model 3: Model 2+ individual variables (insurance premium, occupational status, and number of comorbidities)

Model 4: Model 3+ hospitals and clinics, and urbanization status

^a^ Low: < 33%, Medium: 34–65%; High: ≥ 66%

### Odds ratio of dementia in relation to environmental features and individual characteristics by level of salary-based insurance premium

In Table 6, we used salary-based insurance premium as a proxy of individual socioeconomic status and performed a salary-based insurance premium-stratified analysis to examine the potential effect-modifications by insurance premium on the association between environmental features and the risk of dementia. A significant modification effect of a salary-based insurance premium on the association between “parks, greeneries, and square area”, “playgrounds and sport venues” and the risk of dementia (*p* < 0.0001) was found. For subjects with dependents or salary-based insurance premiums lower than the median, the characteristics of physical and social environment had no significant association with the risk of dementia. But for subjects with salary-based insurance premiums ≥ median, we observed that these cases were less likely to live in townships with a medium density of parks, greeneries, and square area (adjusted OR = 0.91, 95% CI 0.84–0.99) and a high density of playgrounds and sport venues (adjusted OR = 0.85, 95% CI 0.77–0.93) than the controls.

**Table 6:** Odds ratio of dementia in relation to environmental features and individual characteristics in residential townships by level of salary-based insurance premium

Variables	Dependents^C^	<Median^C^	≥Median^C^
**Intercept (SD)**^a^	−0.28(0.13)*	− 0.56(0.15)*	− 0.54 (0.15)*
**Physical environments**			
Parks, greeneries, and square area (ref: Low)^b^			
Medium	0.99 (0.92–1.07)	1.05 (0.94–1.16)	0.91 (0.84–0.99)*
High	1.09 (0.99–1.19)	1.00 (0.89–1.14)	0.97 (0.89–1.07)
Playgrounds and sport venues (ref: Low)^b^			
Medium	0.98 (0.90–1.07)	0.98 (0.87–1.10)	0.95 (0.87–1.04)
High	0.94 (0.84–1.06)	0.97 (0.84–1.13)	0.85 (0.77–0.93)*
Community center (ref: Low)^b^			
Medium	0.96 (0.87–1.06)	0.96 (0.84–1.09)	0.95 (0.87–1.04)
High	1.00 (0.82–1.22)	0.87 (0.67–1.12)	0.99 (0.88–1.12)
**Social environments**			
Median annual family income (ref: Low)^b^			
Medium	1.06 (0.90–1.24)	1.25 (0.99–1.57)	1.06 (0.95–1.17)
High	1.00 (0.84–1.18)	1.15 (0.90–1.46)	1.03 (0.91–1.16)
Illiterate people aged≧65 (ref: Low)^b^			
Medium	0.97 (0.90–1.05)	1.02 (0.92–1.14)	1.03 (0.95–1.13)
High	0.96 (0.84–1.09)	1.06 (0.88–1.27)	1.03 (0.95–1.13)
Elderly living alone (ref: Low)^b^			
Medium	1.02 (0.94–1.10)	1.00 (0.90–1.10)	1.06 (0.97–1.16)
High	1.04 (0.94–1.16)	0.91 (0.79–1.05)	1.07 (0.97–1.19)

^a^*SD* Standard deviation

^b^ Low: < 33%, Medium: 34–65%; High: ≥ 66%

^C^ Based on multilevel logistic regression and adjusted for occupational status, number of comorbidities, parks and greeneries, playgrounds and sport venues, community center, median annual family income, illiterate people aged≧65, elderly living alone, hospitals and clinics, and urbanization status

* *P* < 0.05

## Discussion

This population-based study included a large-scale and representative sample of elderly adults in Taiwan. We found that the risk of dementia significantly decreased by 12% among older adults living in areas with a high density of playgrounds and sport venues and the result did not change in any adjusted model. Also, lower odds of dementia (8%) were found in areas with a medium density of community centers, but the association was not significant after further controlling for individual-level factors. Although higher odds of dementia (14%) were found in areas with high median annual family income, such a significant association did not exist after further adjustment for physical environmental features.

Previous studies on the effects of recreational resources on dementia outcome were limited and mixed. The only two studies on this topic were cross-sectional designs from the UK [7, 8] and one of them found that mixed land use areas (inclusion of residential, commercial and recreational facilities, services and resources) showed no significant effect on prevalent dementia in later life [7]. Despite another UK study showing older adults living in higher mixed land use areas were significantly associated with an approximately 60% decreased odds of dementia [8], it is difficult to interpret the causal-relationship from these findings due to its cross-sectional design.

Our study used a population-based case-control design and found a high density of playgrounds and sport venues (as a proxy for availability of recreational resources) was associated with a 12% decreased odds of dementia in older adults even after controlling for individual factors, health care resources, and urbanization level. The mechanisms by which factors may affect this association remains unclear. However, it has been suggested that the availability of neighborhood spaces for recreational activities significantly promotes adults’ willingness to participate [34], which in turn can lead to older adults spending more time in recreational environments. These conditions are helpful for older adults to increase physical activity [34], social interactions [15] and cognitive stimulations [4], in turn improving mental health [35, 36] and cardiovascular health [37, 38] or enhancing cognitive reserve [19], resulting in a reduced risk of dementia [3, 19].

In addition, animal studies have shown that animals exposed to richer environmental stimulation contribute to neurogenesis via potential ways such as promoting proliferation, astrocyte, and inhibiting cell death [19]. Thus, the availability of playgrounds and sport venues may negatively influence dementia through mechanisms related to participation in recreational environments that could affect dementia directly [19] or lead people to engage in positive health behaviors [15, 19, 34–38].

This study also showed cases of dementia were more likely than controls to live in townships with medium compared with low community center availability. However, this finding is not in agreement with the only published longitudinal cohort study showing that older adults living in neighborhoods with more community resources had slower rates of cognition decline over an 18-year observation period even after adjustment of individual factors [6]. In our study, we did not find any significantly positive association between high density of community centers and dementia. Additionally, the effect of a medium density of community centers on the risk of dementia was no longer significant after adjustment of individual-level characteristics. The reason for this may be much explained by the individual characteristics of the older adults living in these areas, instead of the effects of community centers in local areas. For example, we further found that older adults living in townships with medium community center density have a higher proportion of white- and blue- collar workers (66.9% vs. 41.4%), lower dependence (27.1% vs. 43.6%), and less comorbidities (% with more than two diseases: 24.8% vs. 27.5%) (data not shown). This may explain why these people living in areas with a medium community center density have a lower risk of developing dementia than those living in areas with low community center density.

The effect of green environments was the other environmental effect which has been reported in association with cognition function in older adults [8, 9]. This environmental feature could be helpful in reducing cognitive loading, restoring attention, and thus benefit cognition [4, 9]. However, another study reported that more green environments were significantly associated with increased odds of dementia in later life, possibly because such environments may be associated with isolation, which limits access to local services, resulting in a lack of cognitive stimulation [8]. Our study found no significant association between parks, greeneries, and squares (as a proxy for availability of green environments) and the risk of dementia. This suggests that land use related to parks, greeneries, and squares at the township level in Taiwan doesn’t lead to any visible influences on the risk of dementia among older adults.

Only a few studies investigating the effects of area-based socioeconomic status in relation to the risk of developing dementia have been conducted [20, 21]. A UK cohort study recruiting 6220 nationally representative participants aged 65 years and over with a 12-year follow-up period, investigated the association between an index of multiple deprivation (i.e., a summarized index based on income, employment, education and training, health and disability, barriers to housing and services, living environment, and crime) and dementia incidence. The results showed older adults living in areas in the second-highest quintile of multiple deprivation was associated with an increased risk of dementia (HR = 1.62, 95%CI 1.06–2.46) compared to those living in areas in the least deprived quintile after adjustment of individual factors [20]. Another French study (three-city cohort) including 70 l6 individuals aged 65 years and over with a 12-year observation period also found the risk of developing dementia was positively related to a neighborhood deprivation score in women (HR = 1.29, 95% CI 1.00–1.67) but not in men [21].

The mechanisms by which factors affect the association between area-based socioeconomic status and dementia risk have not been fully identified, but it has been suggested that people living in better socio-economic environments are more exposed to higher densities of recreational resources (e.g., recreation centers, healthy food stores), and social and cultural resources (e.g., libraries, community centers) and thus lead people to engage in positive health behaviors and cognitively stimulating activities [4, 5]. These features could be helpful in reducing the risk of developing dementia. In contrast, people living in deprived areas could be related to poor conditions, lower densities of recreational, social, and cultural resources (less cognitive stimulation) [4], and greater presence of environmental stressors [5]. These latter factors may contribute to an increased risk of dementia.

Contrary to previous studies [20, 21], we found that cases were more likely than controls to live in townships with higher median annual family income, but the significant effect did not exist when physical and social environmental factors were controlled simultaneously. This may suggest that much of the effect of median annual family income is due to the influence of physical and social environments on each other, rather than the features of social environments themselves. It is possible that people with poor cognition may choose to live in areas with better socio-economic environments because they can obtain more health promoting services and resources in the affluent neighborhoods [39, 40]. It is also likely that high median annual family income influences dementia through individual factors because the association between high median annual family income and increased odds of dementia attenuated after controlling for individual factors.

One explanation for this condition could be that there were fewer white-collar workers (13.6% vs. 14.8%) among the dementia cases living in areas with high median annual family income, and they were more likely to have lower insurance premiums (percentage with median and higher insurance premiums: 25.9% vs. 27.8%), and more comorbidities (percentage with more than two diseases: 31.6% vs. 24.3%) than the control group (data not shown). These individual socioeconomic disadvantages may reduce physical activity [41], impair psychological function [42], and increase the risk of chronic disease [43], which contributes to an increased risk of dementia. Alternatively, we speculate that people with poor cognition might choose to live in areas with higher socioeconomic status because there are more medical facilities that can be utilized [44]. Therefore, after further controlling for factors of hospitals and clinics and urbanization level, the effect of high median annual family income on the risk of dementia was weakened; and the significant effect of medium median annual family income on the risk of dementia can be overlooked.

Regarding the effect of area-based education, we found that the adjusted OR of dementia for the exposure of living in townships with a higher percentage of illiterate people aged≧65 did not significantly increase in any adjusted models. Although a previous study reported most domains of cognition function were independently related to social cohesion [10], our study showed no significantly elevated adjusted OR of dementia for the exposure of living in areas with a higher density of elderly living alone. This suggests that the “percentage of illiterate people aged≧65 “and “density of elderly living alone” at the township level in Taiwan are inadequate in detecting associations with the risk of developing dementia among older adults.

As for the effects of salary-based insurance premium at individual level, we observed that salary-based insurance premium at individual level may change the associations of specific features of environments on the risk of dementia. Although no significant difference in the environmental features-specific odds of dementia was observed among subjects with dependents or salary-based insurance premiums lower than the median, the adjusted OR of dementia were significantly negative associated with a medium density of parks, greeneries, and square area (adjusted OR = 0.91, 95% CI 0.84–0.99) and a high density of playgrounds and sport venues (adjusted OR = 0.85, 95% CI 0.77–0.93). The reasons for the results are not clear.

We suspect that increased income status provides better affordable or accessible environmental resources (such as “parks, greeneries, and square area” and “playgrounds and sport venues”), potentially encouraging residents to utilize their environmental resources and resulting in increased cognitive stimulation and social participation. This condition may contribute to the significant effect of “parks, greeneries, and square area” and “playgrounds and sport venues” on the risk of dementia in subjects with salary-based insurance premiums greater than the median rather than dependents or subjects with salary-based insurance premiums lower than the median. More interestingly, we observed that the significantly positive effect of “parks, greeneries, and square area” on the risk of dementia only appeared in the medium density group (adjusted OR = 0.91, 95% CI 0.84–0.99) and not in the high density group, which may have resulted from the different distances to residential parks, greeneries, and square area [32]. A previous study in Taiwan categorized the measures of parks and green spaces into Quartiles and found parks and green spaces in the highest group were farther away from residential areas which may make it hard for the older adults to access. In contrast, the parks and green spaces in the Q3 group were close to residential areas [32].

This study has several strengths. First, we included a large numbers of study subjects by using the Taiwanese NHIRD, which makes the study population highly representative and leaves little room for selection bias. In Taiwan, the NHIRD covers around 99% of the entire population [45]. The NHIA performs expert reviews quarterly on a random sample of 50–100 outpatient and inpatient claims to ensure the accuracy of the claim files [45]. Thus, information obtained from the NHIRD is considered to be complete and accurate. Second, we used a case-control design to collect exposure information more efficient. All exposure information in this study was collected before the first diagnosis of dementia, which is helpful for explaining a causal relationship of the results with fitting temporal inference. Third, the likelihood of prevalence-incidence bias was also largely reduced by using initially diagnosed cases with dementia rather than prevalent cases.

Despite these advantages, this study also has some limitations. First, some environmental factors, such as neighborhood psychosocial disorders (e.g., crime), public transport availability, pollutants, and some individual factors, such as social engagement, smoking status, educational level, physical function and genes were not included in the analysis due to the lack of available data. Although we used occupational status as a proxy for individual educational level and used COPD (i.e., one of the comorbidities) as a proxy for smoking, residual confounding bias is still possible.

Second, the factors potentially associated with dementia analysed in our study included both individual-level and ecological-level variables. Using ecological-level factors to indicate an individual’s exposure status may incur exposure misclassification, which is likely to be non-differential and may result in underestimation of the associations between ecological-level factors and risk of dementia.

Third, we relied on the physician-recorded diagnosis in the medical claims to select dementia cases, which might result in disease misclassification. The medical claims only included medical information for people who sought care for dementia in hospitals or clinics. Therefore, it may have been mixed up with new onset or undiagnosed dementia in the control group. To address this concern, we included solely dementia cases that had at least 3 ambulatory visits with dementia-related diagnosis and the first and last outpatient visits at least 90 days apart to reduce the likelihood of disease misclassification.

Fourth, given the diagnostic procedures of dementia can be different across medical resource and medical care, we adjusted the hospitals and clinics in each township and the level of urbanization to reduce the differences in medical resources and medical care resulting in unequal opportunity to be diagnosed as having dementia among dementia cases.

Fifth, previous studies suggested the pathology often starts at least 10 years before the onset of symptoms of Alzheimer’s disease [46, 47]. Owing to the lack of available nationwide land use data prior to 2006, the retrospective period in our study was limited to 4 years or less. This may not have been adequate for finding longitudinal associations. We only used each subject’s exposure to physical and social environments beginning from the year of 2006, which might have led to some degree of environmental exposure misclassification. However, such exposure misclassification was also likely to be non-differential in the dementia and control groups.

Sixth, our ability to examine the biological gradient effect of environmental features on the risk of dementia was limited by the measures available. We have left this area (such as biological gradient effect) for further investigations.

Finally, information on residential mobility in our study showed about 40% of subjects living in different townships over the 5 years, which may have resulted in relocation bias. To avoid this, we further excluded these subjects in the logistic multilevel regression analysis and found the association between the features of physical and social environments and the risk of dementia was little changed. Thus, relocation bias in our study may be small.

## Conclusions

In conclusion, living in township areas with a high density of playgrounds and sport venues may have a positive effect on decreasing the risk of dementia, independently on other individual and environmental factors. Living in township areas with a medium density of community centers may also decrease the risk of dementia, but such effects are dependent on individual factors. Although higher odds of dementia were found in areas with high median annual family income, such effects are also dependent on physical environmental features. It is suggested that public health interventions may take into account these environmental aspects for preventing the incidence of dementia in older adults. Potential pathways via possible mediators from these environmental characteristics to individual-level incidence of dementia should be further examined in future studies.

## Data Availability

The data that support the findings of this study are available from National Health Insurance Administration Ministry of Health and Welfare in Taiwan but restrictions apply to the availability of these data, which were used under license for the current study, and so are not publicly available. Data are however available from the authors upon reasonable request and with permission of National Health Insurance Administration Ministry of Health and Welfare in Taiwan.

## Abbreviations

COPD: Chronic obstructive pulmonary disease
CI: Confidence intervals
GIS: Geographic information system
HR: Hazard ratio
ICD-9-CM: International Classification of Disease, 9th Revision, Clinical Modification
NHIRD: National Health Insurance Research Data
NHIA: National Health Insurance Administration
ORs: Odds ratios
VIF: Variance inflation factor
